# Colonization of microbiota derived from *Macaca fasciculari*s, Bama miniature pigs, beagle dogs, and C57BL/6J mice alleviates DSS-induced colitis in germ-free mice

**DOI:** 10.1128/spectrum.00388-24

**Published:** 2024-07-11

**Authors:** Yapeng Yang, Zeyue Zhang, Yuqing Wang, Junhua Rao, Jing Sun, Zhimin Wu, Jinhui He, Xiang Tan, Lifeng Liang, Qian Yu, Zhifeng Wu, Huicong Zou, Hang Zhang, Miaomiao Dong, Jixia Zheng, Shuaifei Feng, Wei Cheng, Hong Wei

**Affiliations:** 1 Central Laboratory, Clinical Medicine Scientific and Technical Innovation Park, Shanghai Tenth People’s Hospital, Tongji University, Shanghai, China; 2 State Key Laboratory of Agricultural Microbiology, College of Animal Sciences and Technology, Huazhong Agricultural University, Wuhan, Hubei Province, China; 3 Guangdong Key Laboratory of Animal Conservation and Resource Utilization, Guangdong Public Laboratory of Wild Animal Conservation and Utilization, Institute of Zoology, Guangdong Academy of Sciences, Guangzhou, China; 4 Chongqing Academy of Animal Sciences, Chongqing, China; 5 Key Laboratory of Pig Industry Sciences, Ministry of Agriculture, Chongqing, China; 6 Department of Clinical Veterinary Medicine, College of Veterinary Medicine, Huazhong Agricultural University, Wuhan, China; 7 School of Life Sciences, Inner Mongolia University, Hohhot, China; 8 Precision Medicine Institute, The First Affiliated Hospital, Sun Yat-sen University, Guangzhou, China; 9 Yu‐Yue Pathology Scientific Research Center, Chongqing, China; Huazhong University of Science and Technology, Wuhan, China

**Keywords:** fecal microbiota transplantation, inflammatory bowel disease, metagenome, effective microbe

## Abstract

Fecal microbiota transplantation (FMT) is an innovative and promising treatment for inflammatory bowel disease (IBD), which is related to the capability of FMT to supply functional microorganisms to improve recipient gut health. Numerous studies have highlighted considerable variability in the efficacy of FMT interventions for IBD. Several factors, including the composition of the donor microorganisms, significantly affect the efficacy of FMT in the treatment of IBD. Consequently, identifying the functional microorganisms in the donor is crucial for enhancing the efficacy of FMT. To explore potential common anti-inflammatory bacteria with therapeutic implications for IBD, germ-free (GF) BALB/c mice were pre-colonized with fecal microbiota obtained from diverse donors, including *Macaca fascicularis* (MCC_FMT), Bama miniature pigs (BP_FMT), beagle dogs (BD_FMT), and C57BL/6 J mice (Mice_FMT). Subsequently, mice were treated with dextran sodium sulfate (DSS). As expected, the symptoms of colitis were alleviated by MCC_FMT, BP_FMT, BD_FMT, and Mice_FMT, as demonstrated by the prevention of an elevated disease activity index in mice. Additionally, the utilization of distinct donors protected the intestinal barrier and contributed to the regulation of cytokine homeostasis. Metagenomic sequencing data showed that the microbial community structure and dominant species were significantly different among the four groups, which may be linked to variations in the anti-inflammatory efficacy observed in the respective groups. Notably, *Lactobacillus reuteri* and *Flavonifractor plautii* were consistently present in all four groups. *L. reuteri* exhibited a significant negative correlation with IL-1β, and animal studies further confirmed its efficacy in alleviating IBD, suggesting the presence of common functional bacteria across different donors that exert anti-inflammatory effects. This study provides essential foundational data for the potential clinical applications of FMT.

**IMPORTANCE:**

Despite variations in efficacy observed among donors, numerous studies have underscored the potential of fecal microbiota transplantation (FMT) for managing inflammatory bowel disease (IBD), indicating the presence of shared anti-IBD bacterial species. In the present study, the collective anti-inflammatory efficacy observed across all four donor groups prompted the identification of two common bacterial species using metagenomics. A significant negative correlation between *Lactobacillus reuteri* and IL-1β was revealed. Furthermore, mice gavaged with *L. reuteri* successfully managed the colitis challenge induced by dextran sodium sulfate (DSS), suggesting that *L. reuteri* may act as an efficacious bacterium mediating shared anti-inflammatory effects among variable donors. This finding highlights the utilization of variable donors to screen FMT core bacteria, which may be a novel strategy for developing FMT applications.

## INTRODUCTION

Inflammatory bowel disease (IBD), including ulcerative colitis (UC) and Crohn’s disease (CD), is a chronic inflammatory illness of the gastrointestinal tract caused by abnormal interactions between the gut microbiome and the intestinal immune system (1, 2). IBD affects a substantial number of individuals, and its prevalence is increasing in industrialized nations globally (3, 4). Advances in next-generation sequencing have brought attention to the gut microbiome, shifting the earlier focus from host variables that contributed to the etiology of IBD (5). In particular, in individuals genetically susceptible to IBD, the gut microbiota play a multifaceted role in promoting a dysfunctional immune response (6, 7). Consequently, modulation of the gut microbiota is a promising avenue for IBD treatment (4).

Fecal microbiota transplantation (FMT), which involves the introduction of feces from a healthy donor into a patient’s gastrointestinal tract, represents a strategy for modifying the microbiota to alleviate a broad spectrum of diseases (8–10) and has been applied to treat IBD (11, 12). However, FMT in patients with IBD has yielded variable outcomes (13–19). Unlike pharmaceutical drugs, FMT exhibits substantial variation in the diversity and abundance of microorganisms among donors, which leads to differences between batches of FMT, thereby influencing its overall efficacy (20). Notably, multiple variables, including differences in the composition of donor microorganisms, are associated with distinct clinical outcomes, and the composition of donor flora has emerged as a critical factor influencing the efficacy of FMT in IBD treatment (21). The identification of functional bacteria in the fecal microbiota for the treatment of IBD holds promise for enhancing treatment efficacy. Administering FMT using standardized combinations of functional bacteria represents a potential breakthrough in addressing the inconsistent efficacy of FMT (22). Interestingly, various studies using diverse donors have observed the anti-IBD efficacy of FMT interventions (14, 23–25). These observations implied the potential presence of shared anti-inflammatory bacteria among diverse donors. Consequently, there is a need to investigate the underlying mechanisms contributing to the efficacy of variable donor-based FMT interventions for IBD and screen for common anti-inflammatory bacteria among diverse donors.

Using germ-free (GF) mice with a clear microbiological background helps avoid the interference of native microorganisms, and this approach is particularly important in studies focused on screening functional bacteria for FMT (26). Notably, there is significant variation in the donor flora across species, and the shared flora among different donors is comparatively limited (27–31), which may narrow the scope of screening for commensal anti-inflammatory bacteria. Moreover, the effectiveness of multiple healthy animal flora species in treating IBD remains unclear. It is imperative to use GF IBD models to assess the anti-IBD efficacy of diverse, multispecies donor microbiota and screen for potentially efficacious bacteria shared among the donor flora. To address this, GF BALB/c mice were pre-colonized with the fecal microbiota of donors, including *Macaca fascicularis*, Bama miniature pigs, beagle dogs, and C57BL/6 J mice, followed by the induction of colitis using dextran sodium sulfate (DSS). This study aimed to investigate the efficacy of various FMT donors in IBD treatment and to explore the potential anti-inflammatory commensal bacteria present in various FMT donors. The findings of this study are anticipated to serve as a valuable reference for the clinical application of efficacious FMT bacteria.

## RESULTS

### Microbiota from the four distinct donor groups exhibited variable efficacy in alleviating colitis in mice

**Fig 1:**
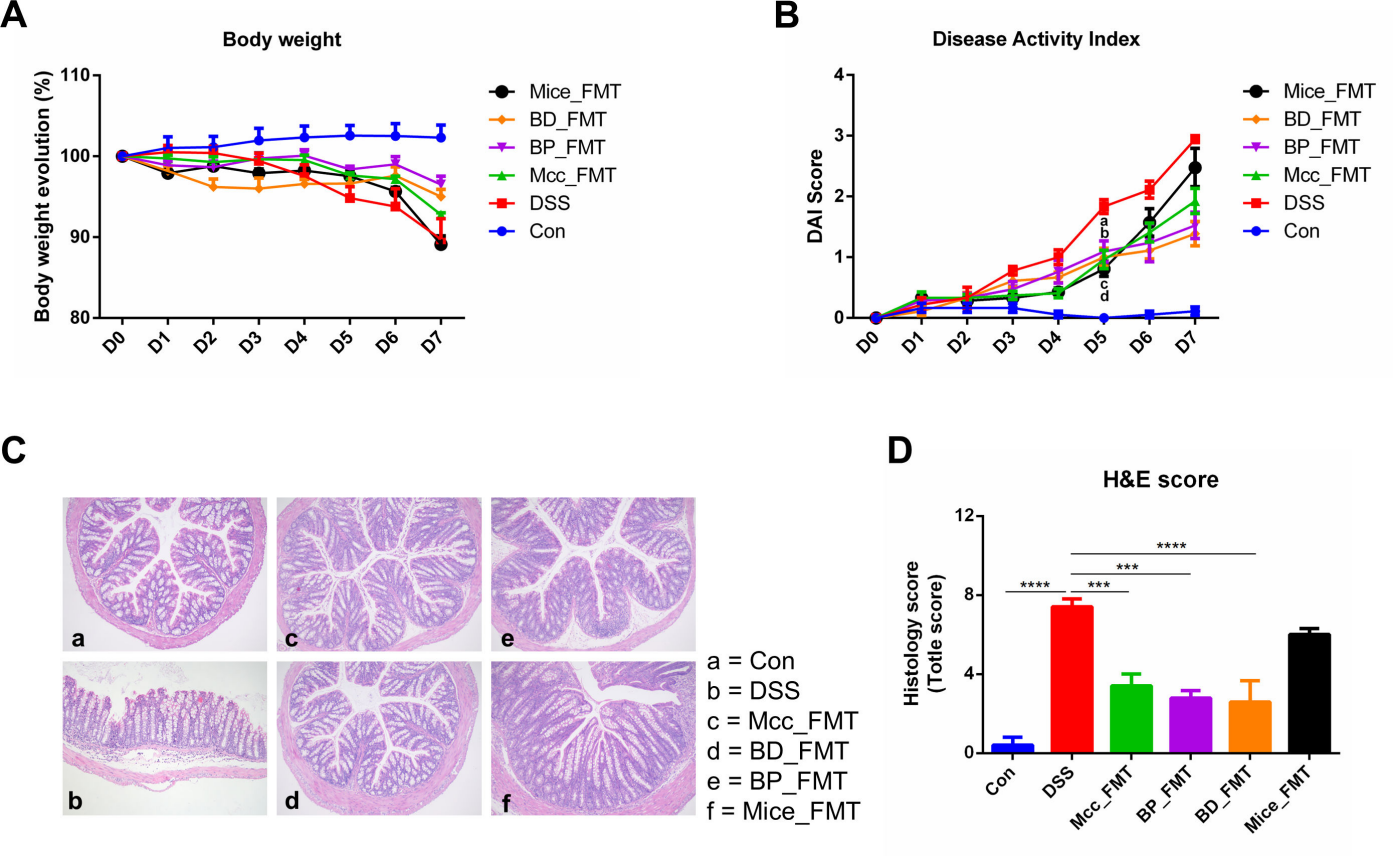
Four groups of FMT mitigated DSS-induced colitis to varying degrees. (**A**) Body weight evolution; (**B**) DAI score; the letters a, b, c, and d represent significant differences in groups Mcc_FMT, BP_FMT, BD_FMT, and Mice_FMT compared to the DSS group, respectively; (**C**) H&E staining of colon tissue (100×); (**D**) Histological score. ***P ≤ 0.001; ****P ≤ 0.0001; data are represented as mean ± SEM.

To investigate the effects of microbiota from donors including *Macaca fascicularis* (Mcc), Bama miniature pigs (BP), beagle dogs (BD), and C57BL/6 J mice (Mice) on colitis, mice were colonized with fecal suspensions of Mcc, BP, BD, Mice, or saline and then treated with 3% DSS. In the later stages of the experiment, mice in the DSS group tended to experience more significant weight loss (Fig. 1A). Mice in all four groups that underwent microbiota transplantation exhibited lower disease activity index (DAI) scores on the fifth day of the experiment (Mcc_FMT vs DSS, *P* = 0.0008; BP_FMT vs DSS, *P* = 0.01; BD_FMT vs DSS, *P* = 0.0042; and Mice_FMT vs DSS, *P* = 0.0002) (Fig. 1B). The results showed that each donor group effectively mitigated the upward trajectory of the DAI in mice. Histological staining showed that DSS treatment resulted in different degrees of histological damage in each group of mice, except in the Mice_FMT group (Mcc_FMT vs DSS, *P* = 0.0009; BP_FMT vs DSS, *P* = 0.0001; BD_FMT vs DSS, *P* < 0.0001; and Mice_FMT vs DSS, *P* = 0.5562) (Fig. 1C and D). In summary, these results suggest that pre-colonization with donor flora from all four groups alleviated DSS-induced colitis, exhibiting distinct effects.

### Effect of the four donor groups on colonic inflammatory markers and cytokines

**Fig 2:**
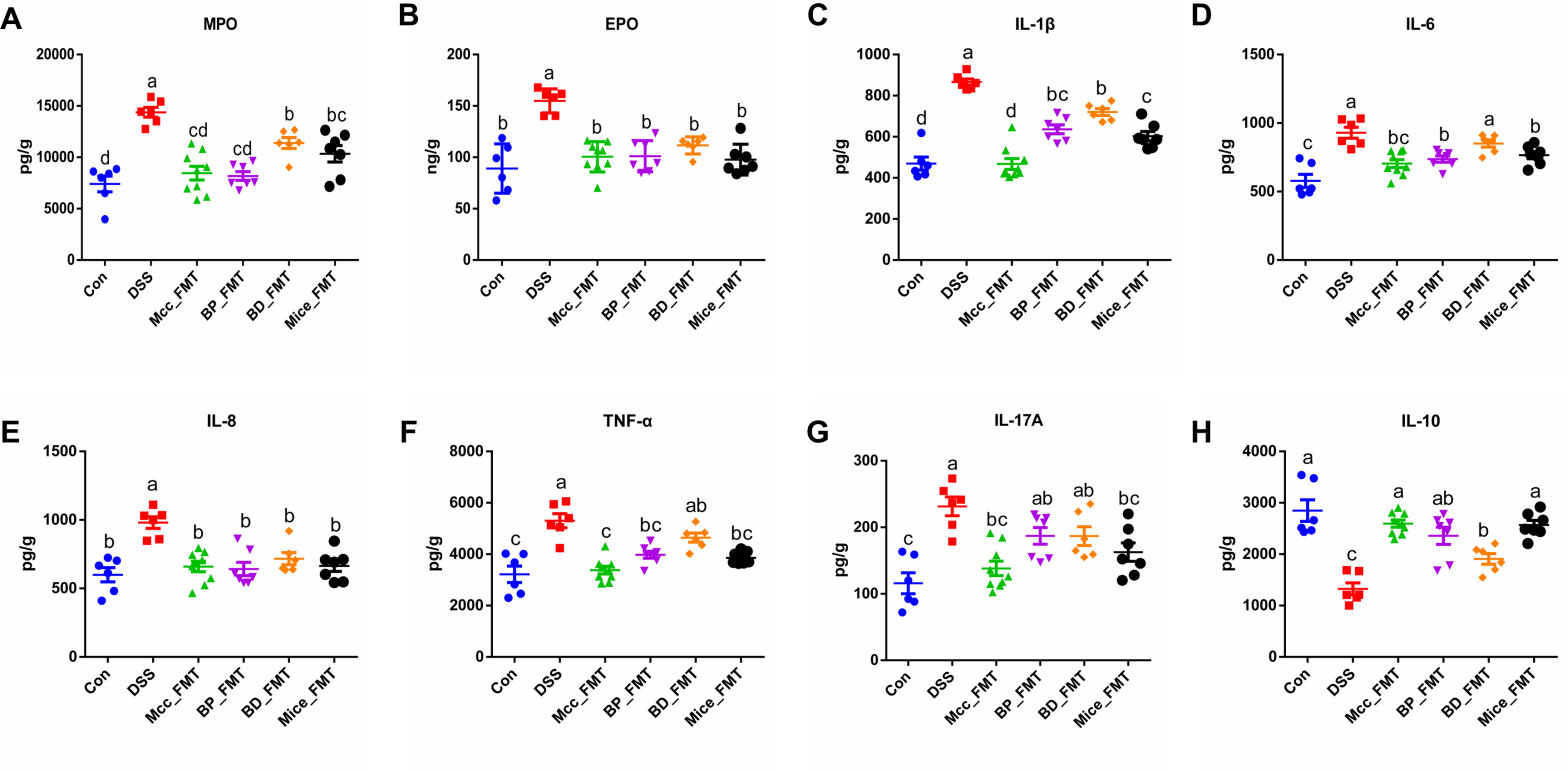
Effect of FMT by four distinct groups on inflammatory cytokine levels in colitis. (**A**) Colonic MPO; (**B**) colonic EPO; (**C**) colonic IL-1β; (**D**) colonic IL-6; (**E**) colonic IL-8; (**F**) colonic TNF-α; (**G**) colonic IL-17A; (**H**) colonic IL-10. Different letters indicate significant differences between groups, while the use of the same letter indicates no significant difference between groups; data are represented as mean ± SEM.

To explore the effects of the donor flora from the four groups on immune homeostasis and inflammatory markers of colitis, the levels of pro-inflammatory cytokines IL-1β, IL-6, IL-8, IL-17A, and TNF-α; anti-inflammatory cytokine IL-10; and colitis markers myeloperoxidase (MPO) and eosinophil peroxidase (EPO) in colonic tissues were measured using ELISA. As shown in Fig. 2, all four groups of donor FMT significantly reduced the levels of MPO, EPO, IL-1β, and IL-8 and increased IL-10 levels in colonic tissues compared to those in the DSS group (Fig. 2A through H, *P* < 0.05). The levels of IL-6, IL-17A, and TNF-α in the BD_FMT group were not significantly different from those in the DSS group (Fig. 2D, F and G), the levels of IL-17A in the BP_FMT group were not significantly different from those in the DSS group (Fig. 2G), whereas the levels of IL-6, IL-17A, and TNF-α in the Mcc_FMT and Mice_FMT groups were significantly lower than those in the DSS group (Fig. 2D, F and G, *P* < 0.05). In summary, pre-colonization with donor flora from the four groups alleviated colitis symptoms to varying degrees by reducing the inflammatory response in mice with colitis.

### Microbiota from the four donor groups improved the intestinal barrier in DSS-induced mice with colitis

**Fig 3:**
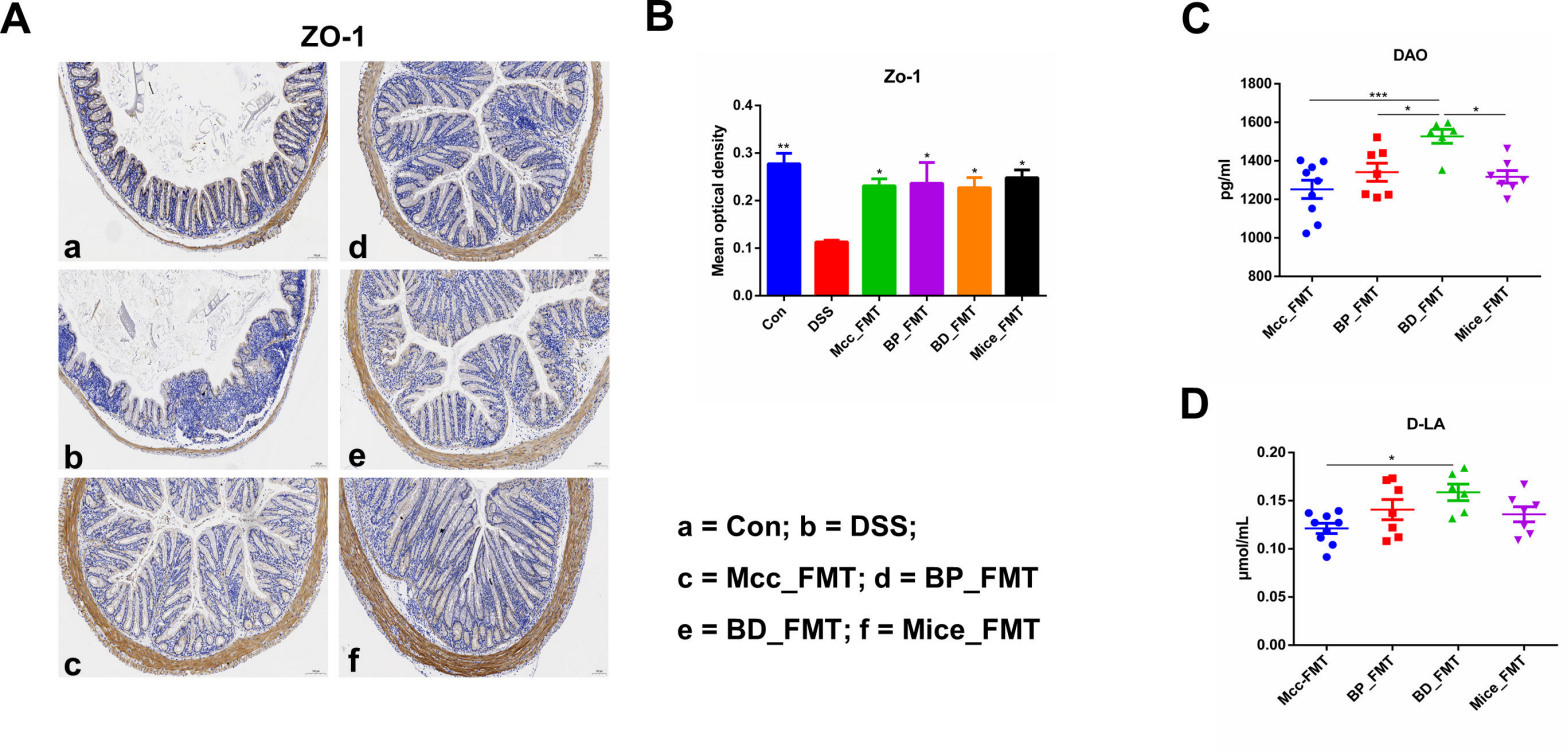
Effect of FMT by four distinct groups on the intestinal barrier in DSS-induced colitis. (**A**) Immunohistochemistry for ZO-1 in each group (100 µm, *n* = 3); (**B**) average optical density and statistical analysis of differences compared to the DSS group. The changes of levels of serum DAO (**C**) and D-lactate (**D**). *P ≤ 0.05, **P ≤ 0.01, and ***P ≤ 0.001; data are represented as mean ± SEM.

IBD is associated with intestinal epithelial barrier dysfunction; therefore, we hypothesized that all donor groups could mitigate DSS-induced damage by protecting the intestinal barrier. The average optical density (AOD) of the tight junction protein ZO-1 was measured in the four groups of mouse colonic tissues using immunohistochemistry (IHC). As shown in Fig. 3A and B, the AOD of colonic ZO-1 was significantly higher in the four FMT groups than in the DSS group (Mcc_FMT vs DSS, *P* = 0.0379; BP_FMT vs DSS, *P* = 0.0286; BD_FMT vs DSS, *P* = 0.0462; and Mice_FMT vs DSS, *P* = 0.0158). To investigate the barrier mechanism underlying the differences in anticolitis efficacy among the four groups, serum diamine oxidase (DAO) and D-lactic acid (D-LA) levels associated with intestinal permeability were evaluated and found to be significantly lower in the MCC_FMT group than in the BD_FMT group, and DAO levels were significantly higher in the BD_FMT group than in the other groups (Fig. 3C and D). In summary, all donor groups alleviated DSS-induced damage by maintaining intestinal barrier integrity, accompanied by different degrees of intestinal barrier protection.

### Microbiota were significantly different between the four groups

**Fig 4:**
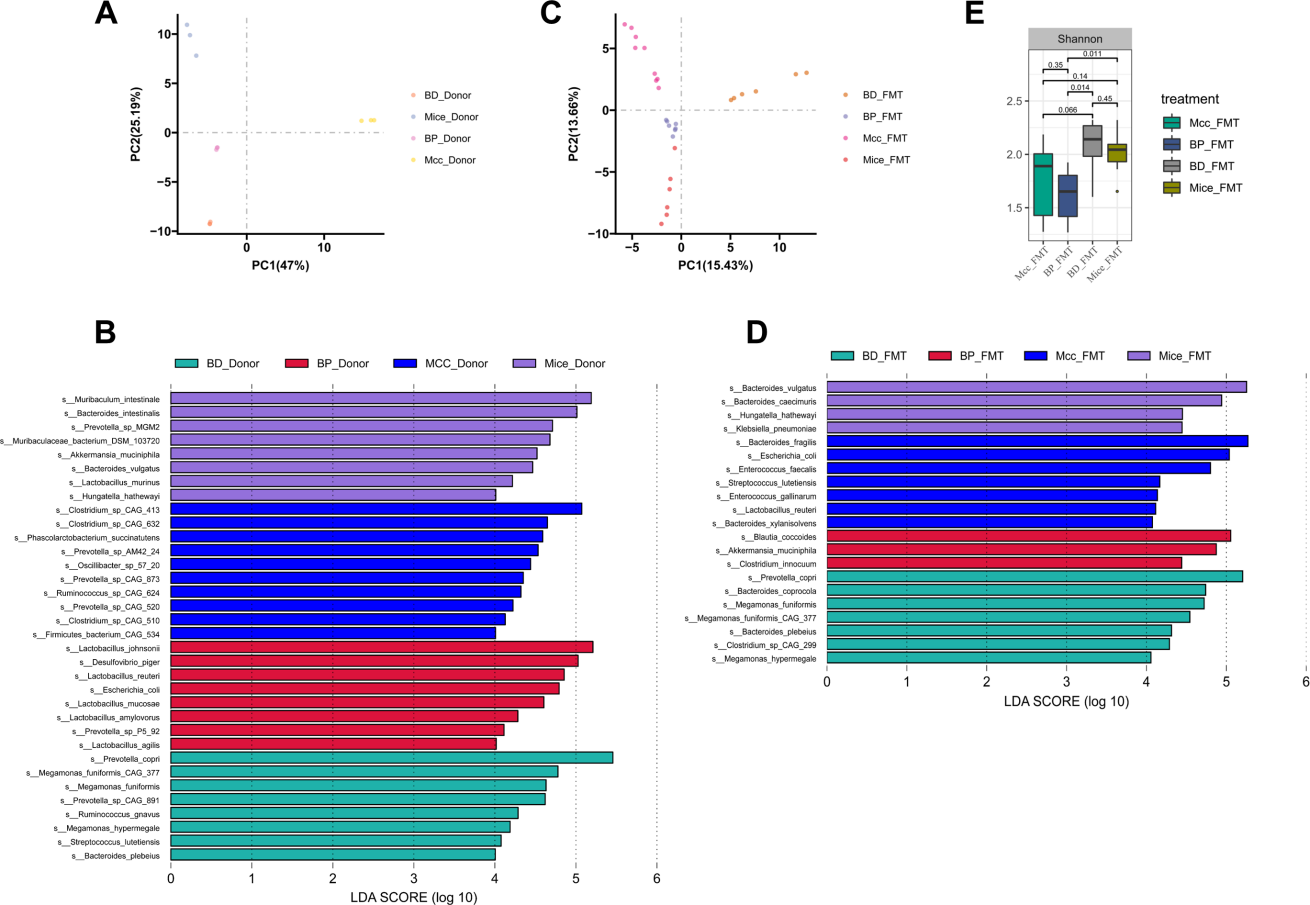
Four groups of microbiological profiles were analyzed. (**A**) PCA for donor groups; (**B**) LEfSe analysis of donor groups; (**C**) PCA for recipient groups; (**D**) LEfSe analysis of recipient groups; (**E**) Alpha diversity of recipient groups, Shannon index.

The initiation and progression of IBD are influenced by the gut microbiota, and the microbiota from the four donor groups exhibited varying efficacies against DSS-induced colitis. The differences in the fecal microbiota between the four groups of donor and recipient mice were analyzed using metagenomics. At the species level, the PCA revealed a significant separation of the microbial community of each donor group from that of the other donor groups (Fig. 4A). Differences in microorganisms among the four groups were analyzed using LEfSe (LDA > 4) to explain the variations in efficacy. In the Mcc donor group, the dominant species were *Clostridium_sp_CAG_413* and *Clostridium_sp_CAG_632*. In the BP donor group, the dominant species were *Lactobacillus_johnsonii*, *Desulfovibrio_piger*, and *Lactobacillus_reuteri*. In the BD donor group, *Prevotella_copri* and *Megamonas_funiformis_CAG_377* were dominant species. Finally, the dominant species in the mouse donor group were *Muribaculum_intestinale* and *Akkermansia_muciniphila* (Fig. 4B).

Among recipient mice, PCA revealed significant differences in the microbial communities among the four recipient groups (Fig. 4C). Furthermore, alpha diversity analysis showed lower diversity in the BP_FMT group than in the BD_FMT and Mice_FMT groups (Shannon index, Fig. 4E). Differences in microbial communities between recipient groups were analyzed using LEfSe (LDA > 4, Fig. 4D), which showed that the dominant species in the Mcc_FMT group included *Bacteroides fragilis*, *Escherichia coli*, *Enterococcus faecalis*, and *Lactobacillus reuteri*. The dominant species in the BP_FMT group were *Blautia coccoides* and *Akkermansia_muciniphila. Prevotella copri*, *Bacteroides coprocola*, and *M. funiformis* were the dominant species in the BD_FMT group. The dominant species in the Mice_FMT group were *Bacteroides_vulgatus* and *Bacteroides_caecimuris*. Overall, the differences in microorganisms were associated with differences in efficacy.

### Screening of potential core anti-inflammatory bacteria based on anti-inflammatory commonalities

**Fig 5:**
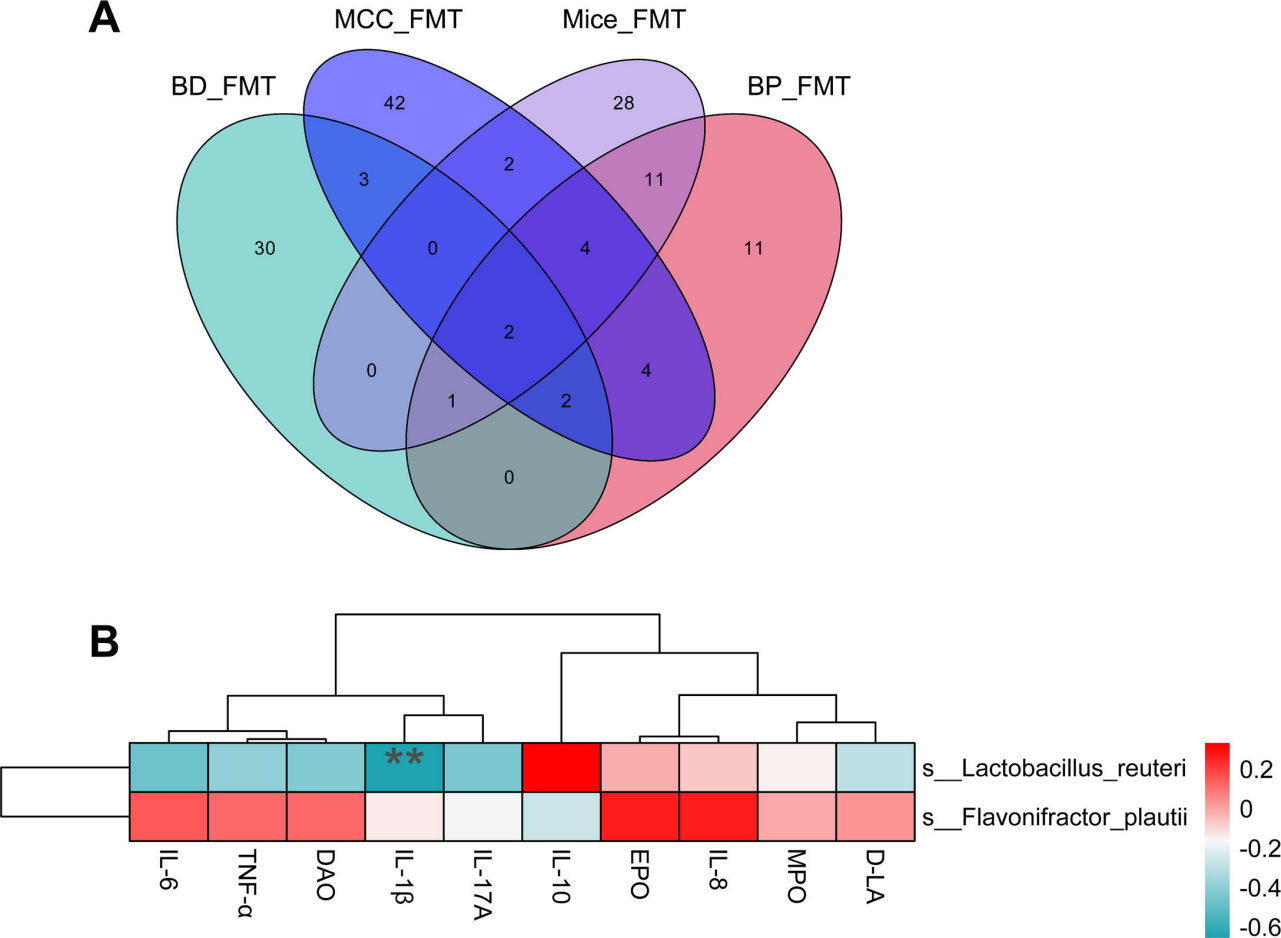
Screening of potential core anti-inflammatory bacteria. (**A**) Venn diagram for four recipient groups; (**B**) correlation analysis of *Lactobacillus reuteri* and *Flavonifractor plautii* bacteria with cytokines. **P ≤ 0.01.

In the present study, all four groups of donor flora alleviated the symptoms induced by DSS in mouse colitis. Moreover, they contributed to the protection of the intestinal barrier and maintenance of immune homeostasis. These findings suggest that there may be bacteria with shared anti-IBD efficacy. Venn diagrams were used to display the potential core anti-inflammatory bacteria shared among the four groups, and both *Lactobacillus reuteri* and *Flavonifractor plautii* were present in the feces of all the FMT groups (Fig. 5A). Subsequently, correlation analysis was conducted between inflammatory cytokine levels and these two potentially efficacious microorganisms. Heatmaps were used to present the results of correlation tests, and *L. reuteri* was significantly negatively correlated with IL-1β (Fig. 5B, *P* < 0.01). This finding suggests an association between *L. reuteri* and the anti-inflammatory efficacy observed in the four groups of FMT donors.

### *L. reuteri* exhibited efficacy in alleviating DSS-induced colitis

**Fig 6:**
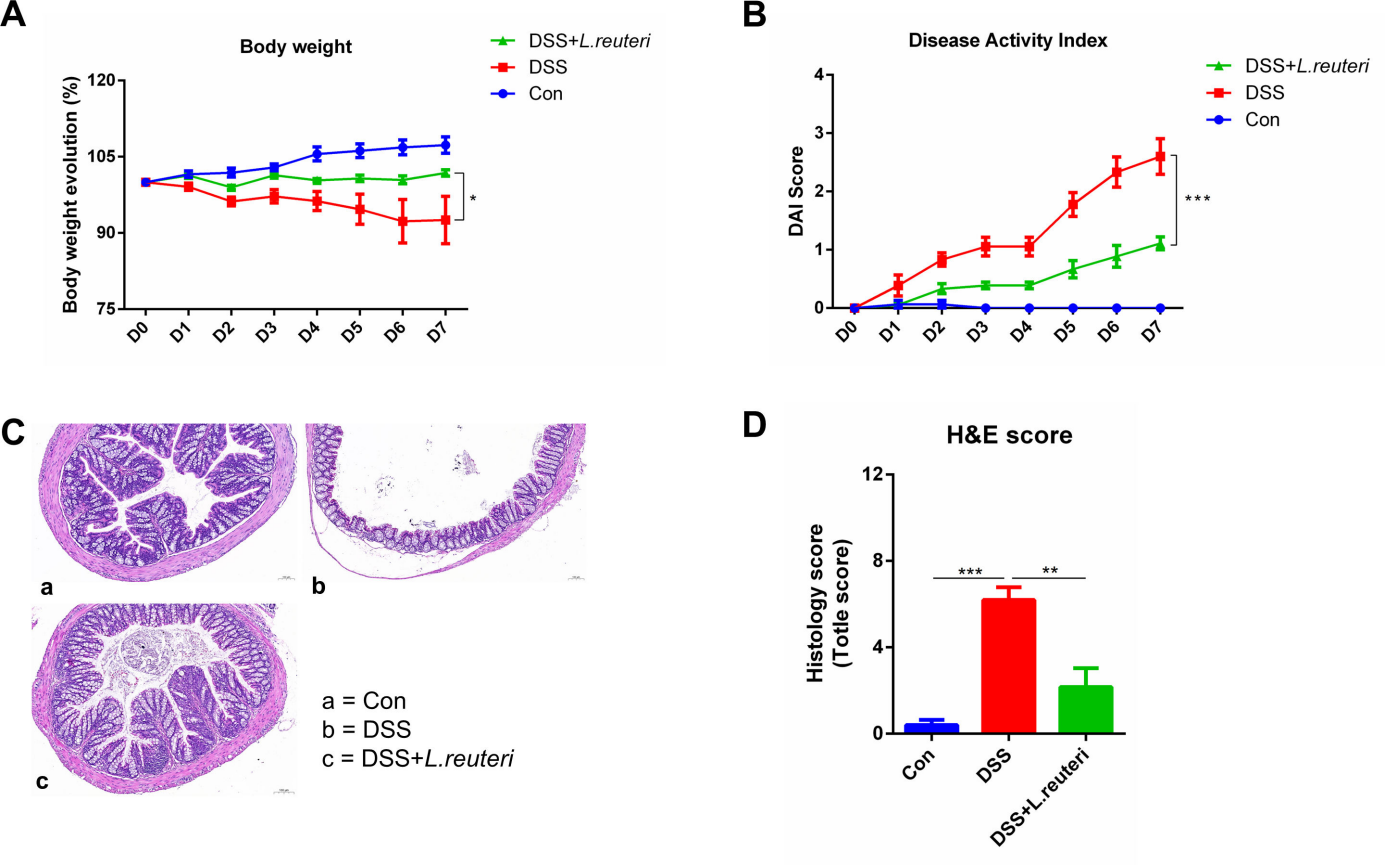
*L. reuteri* exhibits efficacy in alleviating DSS-induced colitis. (**A**) Body weight evolution; (**B**) DAI score; (**C**) H&E staining of colon tissue (100×); (**D**) histological score. *P ≤ 0.05, **P ≤ 0.01, and ****P ≤ 0.0001; data are represented as mean ± SEM, *n* = 5–6 mice per group.

To verify whether *L. reuteri* was effective in alleviating DSS-induced colitis, mice were colonized with *L. reuteri* or saline and then treated with 3% DSS. In the later stages of the experiment, mice in the DSS group tended to experience more significant weight loss, and intervention with *L. reuteri* significantly prevented this weight loss (DSS +*L. reuteri* vs DSS, *P* = 0.0488) (Fig. 6A). Mice in the DSS +*L. reuteri* group exhibited lower DAI scores (DSS +*L. reuteri* vs DSS, *P* = 0.0001) (Fig. 6B). The results showed that *L. reuteri* effectively mitigated the upward trajectory of DAI in the mice. Compared to the DSS group, the DSS +*L. reuteri* group showed reduced histological scores (DSS +*L. reuteri* vs DSS, *P* = 0.0022) (Fig. 6C and D). In summary, these results suggest that pre-colonization with *L. reuteri* could provide *L. reuteri* to alleviate DSS-induced colitis.

## DISCUSSION

IBD is a common condition that significantly affects the quality of life of many individuals (32). Numerous studies have demonstrated alterations in the composition and community of the gut microbiota in IBD patients compared to healthy individuals (33–36). FMT is currently under investigation as an innovative and promising treatment for IBD (11, 12). Although the efficacy of FMT for IBD varies significantly with different donors (21), multiple studies using diverse donors have consistently observed the anti-IBD efficacy of FMT interventions (14, 23–25). This finding suggests the presence of common anti-inflammatory bacteria that may play intervening roles in FMT from different donors. In this current study, to explore the effectiveness of various donor-derived bacteria against DSS-induced colitis and to identify commensal anti-inflammatory bacteria in mice, GF mice with colitis were inoculated with donor flora from different species. The experimental findings demonstrated that FMT from all four groups of donors decreased the increasing DAI in mouse colitis, downregulated the levels of inflammatory biomarkers MPO (37, 38) and EPO (39), and regulated homeostasis of colonic inflammatory cytokines and the intestinal barrier.

Intestinal MPO activity is a reliable indicator of disease severity in IBD (37, 38). In addition, clinical studies have shown that EPO in patients with UC correlates with disease severity. Eosinophil-derived EPO is an important mediator in the development of colitis, and inhibiting EPO activity may potentially slow down the progression of experimental UC (39). In the present study, bacterial fluids from all four donor groups significantly downregulated the levels of two inflammatory markers, indicating the anti-inflammatory efficacy of bacteria derived from different donors. Moreover, dysfunctional and uncontrolled expression of inflammatory cytokines undermines intestinal integrity and contributes to the progression of IBD (40), and intestinal inflammation is regulated by cytokines such as IL-1β, IL-6, IL-8, TNF-α, IL-17A, and IL-10 (40–42). In the current study, compared to the DSS group, diverse donors demonstrated the capacity to regulate distinct inflammatory cytokines. By recruiting granulocytes and activating CD4^+^ T cells in IBD, IL-1β stimulates the production of pro-inflammatory molecules, such as IL-17A, IL-12, and IFN-γ, aggravating intestinal inflammation (43, 44). Conversely, the secretion of the anti-inflammatory cytokine IL-10 plays a pivotal role in ameliorating mucosal damage in IBD and protecting lymphocytes, thereby inhibiting IBD by suppressing host autoimmune responses (45, 46). In addition, FMT from all four groups of donors significantly increased the levels of the anti-inflammatory cytokine IL-10 and decreased the levels of the pro-inflammatory cytokines IL-1β and IL-8. This suggests the existence of a common mechanism of action regulating the balance of inflammatory cytokines in the four donor groups, which is potentially associated to shared core bacteria.

Intestinal epithelial barrier dysfunction is closely associated with IBD (47). ZO-1 serves as a marker of intestinal mechanical barrier integrity (48), and transcription levels of ZO-1 are significantly downregulated in patients with IBD (49). In the present study, all four donor groups showed improved intestinal barrier, suggesting that the intestinal flora can alleviate DSS-induced intestinal barrier damage. The levels of D-LA and DAO, indicators of intestinal permeability, are notably higher in patients with IBD (50). Our results showed that DAO and D-LA levels were higher in the BD_FMT group and lower in the Mcc_FMT and Mice_FMT groups, indicating variations in intestinal permeability following different FMT interventions, which may be related to differences in efficacy and varying degrees of relief from inflammatory cytokine storms.

Gut microbiota significantly influences IBD remission or progression (51). Thus, metagenomic sequencing was used to analyze the microbial composition of the feces in the four donor groups and the corresponding recipient mice to elucidate the microbiome mechanisms underlying the efficacy of FMT. In the present study, discernible variations were observed in the dominant species of the intestinal flora, community structure, and microbial diversity among the four groups, potentially accounting for differences in efficacy. Notably, the abundance of *Bacteroides vulgatus* was positively correlated with significantly increased elastase activity in patients with UC (52). In the present study, LefSe analysis revealed that *B. vulgatus* emerged as the dominant species in the Mice_FMT group, potentially explaining the high histological scores. Additionally, previous findings have demonstrated that administration of *Lactobacillus reuteri* can reverse DSS-induced intestinal mucus thinning and promote the synthesis of tight junction proteins in the colon (53). In the present study, *L. reuteri*, which is presumed to play a pivotal role in the overall efficacy of the intervention, was significantly enriched in the Mcc_FMT recipient group, demonstrating its superior ability to modulate inflammatory cytokines and protect the integrity of the intestinal barrier. The probiotic *Akkermansia muciniphila* (AKK) has attracted attention for its therapeutic potential (54). Notably, AKK was significantly enriched in the BP_FMT group, thereby contributing to the alleviation of symptoms observed in mice in the BP_FMT group. The remission of patients with UC following FMT was correlated with an increased abundance of *Bacteroides plebeius* (55). In the present study, *B. plebeius* was significantly enriched in the BD_FMT group, which was associated with its ability to effectively alleviate colitis symptoms in the recipient mice. *Bacteroides caecimuris* was found to play a key role in mice by affecting community composition and inflammatory responses and involvement in the mediation of short-chain fatty acids (SCFAs). SCFAs have the potential to modulate protective immunity and mitigate tissue inflammation (56–58). Consequently, *B. caecimuris* may act as the dominant species in the mouse FMT group to modulate inflammatory cytokine homeostasis and protect the intestinal barrier. In summary, the microbiological differences between the groups resulted in variations in efficacy.

*L. reuteri* and *Flavonifractor plautii* were present in the feces of recipient mice in all four groups. Mikami *et al*. demonstrated that the administration of *F. plautii* through gavage played a role in inhibiting TNF-α expression in the inflammatory environment of mice, thereby attenuating the inflammatory response in adipose tissue in obese mice (59). The administration of *F. plautii* through gavage resulted in reduced inflammation levels and potent inhibition of IL-17 signaling in DSS-induced mouse colitis, which was associated with lipophosphatidic acid-mediated inhibition of IL-17 by *F. plautii*. Notably, the current study revealed the presence of *F. plautii* in the feces of all four recipient mouse groups, suggesting its role as a core shared bacterium with anti-inflammatory effects. *L. reuteri* plays a crucial role in restoring the balance of intestinal flora and inhibiting diarrhea through a variety of mechanisms, including the production of metabolites, organic acids, and other antagonistic substances that inhibit the growth and reproduction of harmful bacteria and prevent antibiotic-induced diarrhea (60, 61). Treatment with *L. reuteri R2LC* and *4659* significantly reduced intestinal mucosal inflammation in DSS-induced mouse colitis. In addition, *L. reuteri* reversed DSS-induced intestinal mucus thinning symptoms and promoted the synthesis of connective tissue tight junction proteins at the base of colonic crypts (53). In the present study, the shared presence of *L. reuteri* in four distinct groups exhibited a significant negative correlation with IL-1β; this observed negative correlation underscores the potential immunomodulatory effects of *L. reuteri*, contributing to the maintenance of a balanced inflammatory environment. Animal studies further confirmed its efficacy in alleviating IBD.

However, this study has some limitations, and further studies are needed to explore the underlying molecular mechanisms. In addition, further clinical application studies to validate *L. reuteri* for IBD alleviation are needed, and other bacteria effective for FMT should be explored to improve the efficacy of FMT for IBD alleviation.

### Conclusion

Taken together, these results suggest that interventions involving four distinct groups of donors can effectively diminish the susceptibility of mice to DSS-induced colitis by providing anti-inflammatory bacteria, modulating the homeostasis of inflammatory cytokines, and protecting the intestinal mucosal barrier. Notably, the distinct species composition of the "variable FMT" resulted in variations in the efficacy, and two core anti-inflammatory bacteria were identified based on their effectiveness. The efficacy of *L. reuteri* in relieving IBD was verified. This indicates the possibility of identifying shared anti-inflammatory bacteria for IBD by leveraging the "variable FMT" approach, which provides basic data to support the development of FMT in IBD treatment.

## MATERIALS AND METHODS

### FMT preparation

Fresh fecal samples from *Macaca fascicularis* (5-year-old, Guangzhou Xiangguan Biotechnology Co., Ltd, *n* = 6), Bama-miniature pigs (1-year-old, Chongqing Academy of Animal Science, *n* = 3), Beagle dogs (6-month-old, Huazhong Agricultural University, *n* = 6), and C57BL/6 J mice (8-week-old, Experimental Animal Center of Huazhong Agricultural University, *n* = 20) were collected using a sterile fecal collector, transported to the laboratory under dry ice conditions, and the feces from the interior of the mid-section were homogenized under anerobic conditions (80% N_2_, 10% H_2_, and 10% CO_2_) and mixed thoroughly with saline glycerol buffer (15% glycerol concentration) at a ratio of 1:10 (m/v). Following complete dissolution, the samples underwent rough filtration via a sterile gauze and a final filtration via a 100-µm membrane (62).

### Experimental animals and treatments

**Fig 7:**
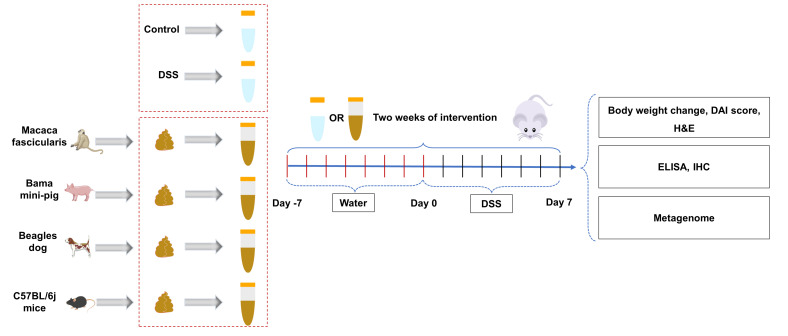
Schematic design. The objective of the experiment was to investigate the impact of FMT from different donors on IBD; the mice were randomly divided into the control group (*n* = 6), DSS group (*n* = 6), MCC_FMT group (*n* = 9), BP_FMT group (*n* = 7), BD_FMT group (*n* = 6), and Mice_FMT group (*n* = 7). Recipient mice were administered 100 µL of the bacterial solution or saline orally daily, and then mice were treated with 3% DSS. During the experimental period, body weights, stool traits, and fecal bleeding were quantified in the mice. At the end of the experiment, mice were euthanized, and colon and fecal samples were collected for subsequent analysis.

Eight- to ten-week-old female GF BALB/c mice were acquired from Huazhong Agricultural University’s Germ-Free Animal Platform. Mice were housed in a sterile environment (temperature 25±2°C; relative humidity 45%–60%; photoperiod 12 hours/day; light hours 06:30–18:30) and had free access to sterilized food and water. The objective of the experiment was to investigate the impact of FMT from different donors on IBD; the mice were randomly divided into the control group (*n* = 6), DSS group (*n* = 6), MCC_FMT group (*n* = 9), BP_FMT group (*n* = 7), BD_FMT group (*n* = 6), and Mice_FMT group (*n* = 7). Recipient mice were administered 100 µL of bacterial solution or saline orally daily, and then mice were treated with 3% DSS. The experimental grouping and timeline are shown in Fig. 7. Throughout the experiment, body weight was measured every day in order to determine the difference between the weight on day 0 and day of measurement (63). Upon completion of the experiment, the feces samples of the mice were collected inside the isolator, and the mice were euthanized. All experimental methods were performed according to the Huazhong Agricultural University of Health Guide for the Care and Use of Laboratory Animals. The animal experiment ethics numbers for this study are HZAUMO-2024–0048 and HZAUMO-2024–0109.

### Culture of *L. reuteri* and its efficacy verification

*Lactobacillus reuteri NCU-15* (*L. reuteri*) was obtained from the laboratory of Tingtao Chen at Nanchang University, and *L. reuteri* was cultured in modified MRS medium at 37°C. To verify whether *L. reuteri* exhibits efficacy in alleviating DSS-induced colitis, the mice were randomly divided into Control group, DSS group, and DSS +*L. reuteri* group (five to six mice per group). Recipient mice were administered 100 µL of the bacterial solution (10^9^ CFU/mL) or saline orally daily, and then mice were treated with 3% DSS.

### Disease Activity ndex

As seen in Table 1, the DAI comprises stool traits, fecal bleeding score, and weight loss score (64, 65). The DAI was calculated by averaging the three scores and measuring the following: weight change (no change = 0; 1%–5% = 1; 5%–10% = 2; 10%–15% = 3; >15% = 4), fecal bleeding score (normal colored stool = 0; brown stool = 1; red stool = 2; bloody stool = 3; heavy bleeding = 4), and stool traits (normal stool, good shape = 0; soft stool, soft stool adhering to the anus = 1–2; diarrhea, adherent anal = 3–4).

**TABLE 1:** Disease Activity Index^*a*^

Score	Weight loss (%)	Stool consistency	Bloody stool score
0	None	Normal	Normal colored stool
1	1–5	Loose stool	Brown stool
2	5–10	Loose stool	Reddish stool
3	10–15	Diarrhea	Bloody stool
4	> 15	Diarrhea	Gross bleeding

^*a*^ Disease Activity Index (DAI), mean score of weight loss, stool consistency, and bloody stool score.

### Mice colon histologic analysis

The distal colon segments from each group of mice were fixed with 4% paraformaldehyde, paraffin-embedded, and cut into 4-μm-thick slices. Slices were stained with hematoxylin–eosin (H&E) and immunohistochemistry (IHC), and images were acquired under a microscope (Nikon Eclipse 80i, Japan). The H&E-stained slices were investigated for inflammatory cell infiltration and tissue damage, and intestinal damage was evaluated for infection degree, extent of infection, crypt damage, and mucosal involvement (66) (see Table 2). The expression of ZO-1 (Proteintech Group, Inc. 21773–1-AP) was detected by immunohistochemistry using Image Pro Plus 6.0 (Media Cybernetics, Inc.) for statistical analysis of mean optical density.

**TABLE 2:** Histological grading of colitis

Grade	Inflammation	Extent	Crypt damage	Percent involvement
0	None		None	0
1	Slight	Mucosa	Basal 1/3 damage	1%–33%
2	Moderate	Mucosa and submucosa	Basal 2/3 damage	34%–66%
3	Severe	Transmural	Entire crypt and epithelium lost	67%–100%

### Enzyme-linked immunosorbent assay (ELISA)

The kits were purchased from Shanghai Enzyme-linked Biotechnology Co., Ltd. (Shanghai, China). ELISA was used to determine the concentrations of IL-1β (ml063132), IL-6 (ml002293), IL-8 (ml001856), IL-10 (ml002285), IL-17A (ml037864), TNF-α (ml002095), myeloperoxidase (MPO) (ml002070), eosinophil peroxidase (EPO) (ml769125), and serum diamine oxidase (DAO) (ml002070) according to the manufacturer’s instructions. Tissue processing for ELISAs are as follows: 1 g of the tissue sample was weighed, and then 9 mL of PBS (pH 7.2–7.4) was added to homogenize the sample. Centrifuge for about 20 mins (2,000–3,000 rpm), and carefully collect the supernatant for testing. Briefly, the diluent buffer from the kit was used to dilute both standards and samples. A microtiter plate with an antibody precoated in each well was then filled with 100 µL of the sample or standard in duplicate. Diluent buffer was used as a negative control. The plates were incubated at 37°C for 2 hours. Following incubation, each well was filled with 100 µL of biotin antibody after the liquid was removed and incubated for 1 hour at 37°C. Wash buffer of 200 µL was used to wash the wells three times. Next, 100 µL of horseradish peroxidase–avidin was added to each well and incubated for an hour at 37°C. Following the final wash, 90 µL of the supplied TMB substrate was added, and the mixture was incubated at 37°C in the dark for 30 minutes. The reaction was stopped using 50 µL of the supplied stop solution. With a plate reader (BioTek Instruments, Inc.) to measure absorbance at 450 nm, the standard curve was used to determine the levels of cytokines in the samples.

### D-lactate measurements

Permeability tests were performed on the serum levels of DAO and D-lactic acid (D-LA). D-LA (ml158174) was measured using spectrophotometry with a D-Lactic acid detection kit (Shanghai Enzyme-linked Biotechnology Co. Ltd.). Briefly, the visible spectrophotometer (Shanghai Enzyme-linked Biotechnology Co. Ltd.) is preheated for more than 30 minutes, the wavelength is adjusted to 450 nm, and zeroed with distilled water. As per the manufacturer’s instructions, the samples and reagents were subsequently added to a 1-mL glass cuvette. The mixture was then mixed, and the reaction was allowed to proceed for 30 minutes at 37°C in the dark. At 450 nm, the absorbance value was measured. The standard curve was used to calculate the D-LA levels in the sample.

### Fecal microbiota analysis

Following collection of the mouse feces samples in a sterile cage within the isolator, the samples were transported on dry ice for sequencing, and DNA extraction was done using the cetyltrimethylammonium ammonium bromide (CTAB) method. For the purpose of preparing the DNA samples, an input material of 1 µg DNA per sample (with an OD value ranging from 1.8 to 2.0) was utilized. In accordance with the manufacturer’s instructions, sequencing libraries were generated using the NEBNext Ultra DNA Library Prep Kit for Illumina (NEB, USA), and index codes were added to each sample to attribute its sequences. Briefly, the DNA sample was fragmented by sonication to a size of 350 bp, and then DNA fragments were end-polished, A-tailed, and ligated with the full-length adapter for Illumina sequencing with further PCR amplification. Lastly, PCR products were purified (AMPure XP system), libraries were examined for size distribution using an Agilent 2100 Bioanalyzer, and real-time PCR was used to quantify the results. Following the manufacturer’s instructions, the index-coded samples were clustered using a cBot Cluster Generation System. After cluster generation, the library preparations were sequenced on an Illumina HiSeq platform, and paired-end reads were generated. The reads of the filtering host were compared with the Chocophlan (Version MPa _ V30 _ Chocophlan _ 201901) database using MetaPhlAn3 (version 3.0.7) software to determine the species abundance information for further analysis. The data were imported into Rstudio (4.1.1) and plotted using ggplot2 (3.3.5) for the mapping of alpha diversity and principal component analysis. The results were analyzed using the Kruskal–Wallis test for multisample comparisons, and the BH method was used for multiple-testing correction of the *P*-value. Distinctive and shared features, Spearman rank correlation (FDR correction), and linear discriminant analysis effect size (LEfSe, *P* < 0.05, LDA > 4.0) were completed using Wekemo Bioincloud (https://www.bioincloud.tech).

### Statistical methods

The data were analyzed using GraphPad Prism 6 (GraphPad Software, San Diego, CA). Data from more than two groups were compared using one-way ANOVA, followed by Tukey’s multiple comparison tests. All data were represented as means ± SEM. *P* ≤ 0.05 was considered statistically significant.

## Data Availability

The raw data supporting the conclusions of this manuscript will be made available by the authors, without undue reservation. The data sets generated and analyzed during the current study were available in the NCBI repository, submission ID: SUB14342893, BioProject: PRJNA1092791.
